# A 4 mm^2^ Double Differential Torsional MEMS Accelerometer Based on a Double-Beam Configuration

**DOI:** 10.3390/s17102264

**Published:** 2017-10-02

**Authors:** Tongqiao Miao, Dingbang Xiao, Qingsong Li, Zhanqiang Hou, Xuezhong Wu

**Affiliations:** College of Mechatronics Engineering and Automation, National University of DefenseTechnology, Changsha 410073, China; tongqiaomiao@163.com (T.M.); liqingsong336@163.com (Q.L.); houzhanqiang@nudt.edu.cn (Z.H.)

**Keywords:** double differential, torsional, MEMS, accelerometer, double-beam, temperature robustness

## Abstract

This paper reports the design and simulation of a 4 mm^2^ double differential torsional MEMS accelerometer based on a double-beam configuration. Based on the structure of conventional torsional accelerometers, normally composed of one pair of proof masses and one torsional beam, this work explores the double differential configuration: a torsional accelerometer with two pairs of unbalanced proof masses rotating in reverse. Also, the torsional beam is designed as a double-beam structure, which is a symmetrical structure formed by two torsional beams separated by a certain distance. The device area of the novel accelerometer is more than 50 times smaller than that of a traditional double differential torsional MEMS accelerometer. The FEM simulation results demonstrate that the smaller device does not sacrifice other specifications, such as mechanical sensitivity, nonlinearity and temperature robustness. The mechanical sensitivity and nonlinearity of a ±15 g measuring range is 59.4 fF/g and 0.88%, respectively. Compared with traditional single-beam silicon structures, the novel structure can achieve lower maximum principle stress in critical regions and reduce the possibility of failure when high-g acceleration loading is applied along all three axes. The mechanical noise equivalent acceleration is about $0.13\;\mathrm{mg}/\sqrt{\mathrm{Hz}}$ in the theoretical calculations and the offset temperature coefficient is 0.25 mg/℃ in the full temperature range of −40 ℃ to 60 ℃.

## 1. Introduction

Year after year the MEMS market is growing faster than the average semiconductor industry and accelerometers are widely used in most MEMS products [1,2,3,4]. MEMS accelerometers are widely used in consumer electronics, such as cell phones, portable gaming consoles, cameras and pedometers to detect vibration or tilt motion [5]. Many kinds of MEMS accelerometers have been reported recently, such as capacitive [2,3,4], resonant [6,7,8], optical [9], and piezoresistive accelerometers [10] and so on. Although a resonant accelerometer achieved μg level of bias stability in [6] and an optical accelerometer achieved tens of μg level resolution in [9], which are better than capacitive accelerometers, the structures are more complex to fabricate and the cost is higher [2,3,4,5,6,7,8,9]. The piezoresistive accelerometer has a large measurement range from 0.25 g to 25,000 g and it is easy to fabricate according to [10], but the temperature robustness is the main challenge. This paper mainly focuses on the study of capacitive accelerometers. Among capacitive accelerometers, the torsional accelerometer is one of the most popular structures for its simple design and low cost, but challenges remain in increasing their bias stability and temperature robustness [11], which are the main obstacles that need to be overcome for inertial navigation systems [12]. Some studies to solve these problems have been reported. The typical methods are temperature compensation with readout circuits [13] or using a thermostatic package [14]. Some other methods based on structure design and package stress isolation have also been proposed, such as a four point supporting frame [15], a highly symmetrical spring-mass structure [16], soft adhesive attachment [17], and special fabrication processes [18], but the methods are complicated and expensive [15,16,17,18].

Inspired by the high performance butterfly gyroscope [19,20] and the differential vibrating beam accelerometer [21], our team has designed and reported two types of double differential torsional MEMS accelerometers based on single-beam structures [22,23]. To further improve the performance and promote the commercial value, this paper presents a novel double-beam double differential torsional MEMS accelerometer of only 4 mm^2^, which utilizes a double differential configuration composed of four proof masses split into two pairs. The double differential arithmetic is introduced for matching the double differential configuration to reduce the output drift to 0.25 mg/℃ in the full temperature range of −40 ℃ to 60 ℃. Compared with traditional single-beam silicon structures, the novel structure can achieve a lower maximum principle stress in critical regions and reduce the possibility of failure by changing the position of critical regions. Although the device area of our novel accelerometer is more than 50 times smaller than that of traditional double differential torsional MEMS accelerometer [22,23], the novel device does not sacrifice other specifications, such as sensitivity and nonlinearity, which are 59.4 fF/g and 0.88%, respectively, for a measuring range of ±15 g. The mechanical noise is analyzed theoretically and the calculated equivalent acceleration is about $0.13\;\mathrm{mg}/\sqrt{\mathrm{Hz}}$ in the theoretical calculation. The fabrication tolerance is also analyzed and the fabrication process is introduced. Afterwards, a FEM simulation by the COMSOL software verifies all the results presented in this paper. The remainder of the paper is hence organized as follows: Section 2 provides a description of the structure design and theoretical consideration of the novel structure. Then, Section 3 collects the results of our simulation analysis of mechanical sensitivity, nonlinearity, shock resistant, tolerance in fabrication and temperature robustness. Next, Section 4 provides the design of the fabrication process. Finally, some concluding remarks and proposals for future works are collected in Section 5.

## 2. Structure Design and Theoretical Considerations

### 2.1. Description and Working Principle

**Figure 1:**
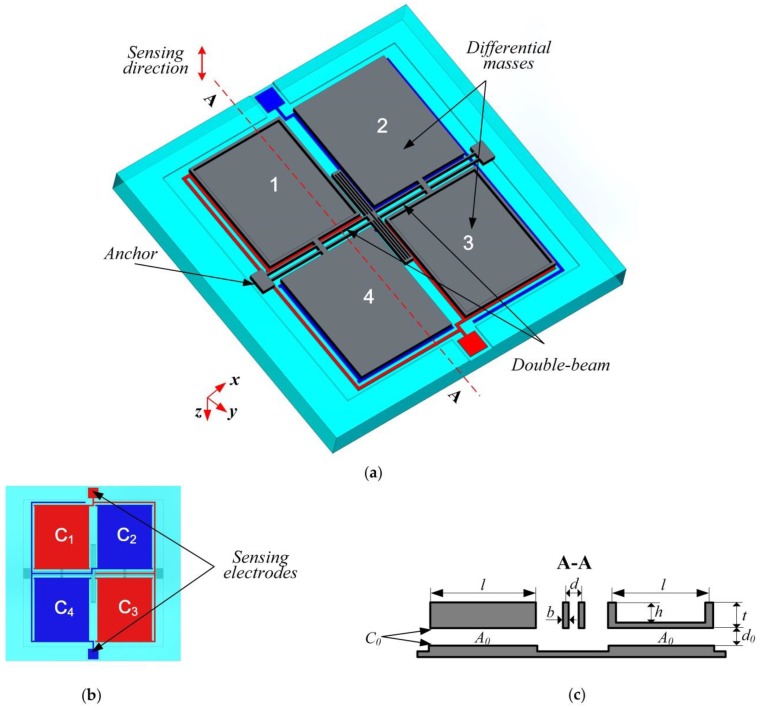
Design model of the accelerometer: (**a**) Overview of the device; (**b**) Back view of the device; (**c**) The cross section of the silicon structure.

As Figure 1 presents, the double differential capacitive micro-accelerometer presented in this paper basically consists of two parts: a silicon substrate and a silicon structure bonded onto it. The overview of the device shows that the silicon structure is mainly composed of four proof masses, a double-beam and a stress-released structure. The arrow along the *z*-axis indicates the sensing direction of the device. The double-beam is the torsional beam of the proof masses formed by silicon dry etching. Four rotatable proof masses can be divided into two pairs and each pair forms a torsional structure. We define that the first pair of masses are mass 1 and mass 4 and the second pair of masses are mass 2 and mass 3. Mass 1 and mass 3 are etched with the same curved depth to realize the unbalanced masses of each pair. The cross section of the silicon structure is presented in Figure 1c and the geometrical parameters of the novel structure are listed in Table 1. Taking navigation application requirements into consideration, the range of this accelerometer is designed as ±15 g. An open-loop circuit is utilized to simplify the readout circuit. The capacitive gap is designed as 2 μm to realize excellent sensitivity, non-linearity and signal to noise ratio properties. Only the capacitive gap parameter needs to be changed when different sensitivities and measuring ranges are required in other applications.

**Figure 2:**
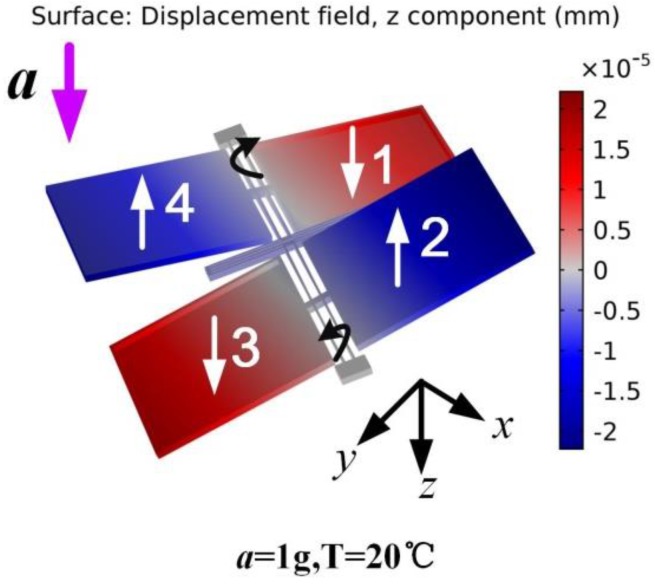
FEM simulation when 1 g acceleration is applied along the sensing direction, the positive *z* direction, at room temperature. The color shows the displacement of the silicon structure compared with the initial position along the sensing direction (*z* direction).

It can be seen that the arrow along the *z*-axis indicates the sensing direction of device. When an acceleration along the sensing direction is applied, the two torsional structures will rotate reversely because of the unbalanced masses of each pair. The working principle is shown in the form of simulation result in Figure 2.

On the silicon substrate, there are four electrodes made to form four capacitors with silicon planes of four masses. Thus, the acceleration along the sensing direction can be calculated by double differential capacitance signals:

(1) $$a\propto \left(\Delta C_{1}-\Delta C_{4}\right)-\left(\Delta C_{2}-\Delta C_{3}\right)=\left(\Delta C_{1}+\Delta C_{3}\right)-\left(\Delta C_{2}+\Delta C_{4}\right)$$

Herein, $\Delta C_{i},\;\;i=1\sim 4\;$ is the changed capacitance of the capacitor formed of the mass *i* and the electrode below it. The measurement of the capacitances addition in Equation (1) is realized by the connection of the diagonal electrodes on the silicon substrate, as shown in Figure 1b. Thus, the output signals of two sensing electrodes are addition of capacitance 1 and 3, 2 and 4, respectively.

**Figure 3:**
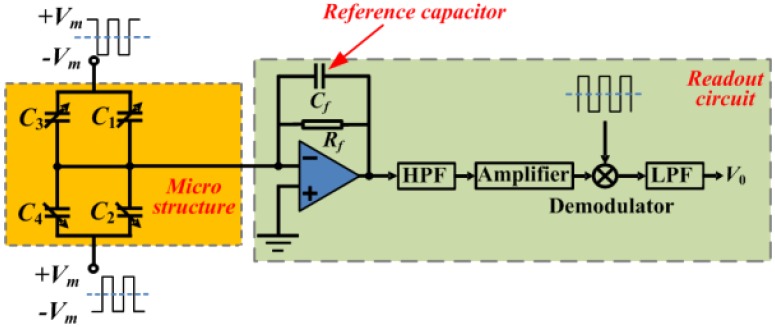
Schematic of the readout circuit.

The schematic of readout circuit is shown in Figure 3. It mainly consists of the sensing element, the charge amplifier, the high pass filter, the second amplifier, the demodulator and the low pass filter.

Thus, the total output voltage can be calculated as:

(2) $$V_{0}=kV_{m}\frac{\left(C_{1}+C_{3}\right)-\left(C_{2}+C_{4}\right)}{C_{f}}$$

where k is the gain of the signal amplifier and $C_{f}$ is the reference capacitance.

### 2.2. Theoretical Consideration

**Figure 4:**
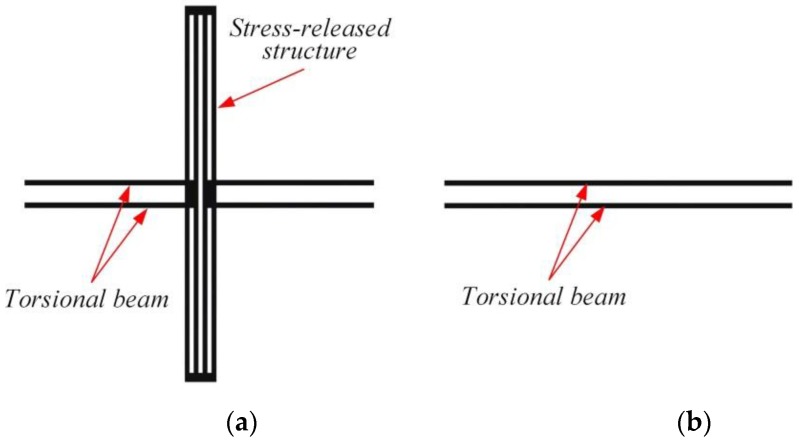
Simplification of stress-released structure: (**a**) Original model; (**b**) Simplified model.

The original stress-released structure is shown in Figure 4a. As the width of the stress-released structure is only 2% of the length of torsional beam, the design of the stress-released structure has very little influence on the stiffness of the torsional beam. Thus, the stress-release-structure can be ignored and the simplified model is shown in Figure 4b. Since the mass of the beam is very small compared to that of the proof mass in this structure, it is neglected in the theoretical analysis.

**Figure 5:**
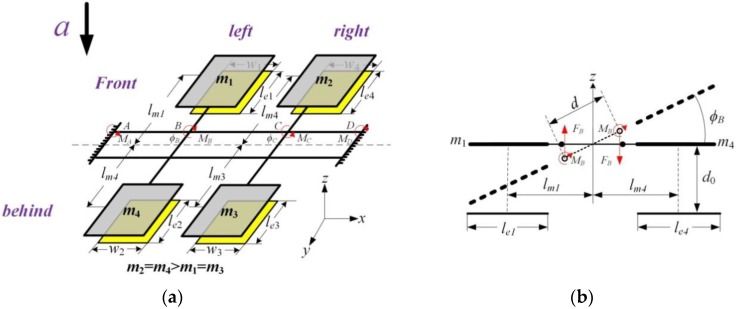
Mathematical model of double differential torsional capacitive accelerometer based on double-beam: (**a**) Torque model of the front torsional beam; (**b**) Mechanical model of the left torsional structure.

Thus, the mathematical model of double differential torsional capacitive accelerometer based on double-beam is shown in Figure 4. Because of the symmetrical characteristic of the structure, two torsional beams and two torsional structures have similar force states, respectively. This paper chooses one of each to analyze the torsional stiffness and the mechanical sensitivity of the double differential torsional capacitive accelerometer. As Figure 5a presents, to make the structure symmetrical, the parameters of the device are set as:

(3) $$m_{2}=m_{4}>m_{1}=m_{3}$$

(4) $$l_{m1}=l_{m2}=l_{m3}=l_{m4}=l_{m}$$

(5) $$l_{e1}=l_{e2}=l_{e3}=l_{e4}=l_{e}$$

(6) $$w_{1}=w_{2}=w_{3}=w_{4}=w$$

(7) $$l_{AB}=l_{CD}=l_{e}$$

(8) $$l_{BC}=l_{s}=kl_{e}$$

(9) $$l_{AD}=L=l_{s}+2l_{e}=(k+2)l_{e}$$

According to the calculation formula of twist angle, the torques of the front torsional beam and the twist angles of both torsional structures can be calculated as:

(10) $$M_{A}=M_{D}=\frac{k}{k+2}M_{B}=\frac{k}{k+2}M_{C}$$

(11) $$\phi_{B}=\phi_{C}=\frac{k}{{\left(k+2\right)}^{2}}\frac{M_{B}L}{GI_{t}}=\frac{k}{{\left(k+2\right)}^{2}}\frac{M_{C}L}{GI_{t}}$$

herein, *G* is the shear modulus of silicon, and $I_{t}$ is torsional moment of inertia which is relative with the cross section. $\phi_{S}$ can be expressed as:

(12) $$\phi_{S}=\phi_{B}=\phi_{C}=\frac{k}{{\left(k+2\right)}^{2}}\frac{M_{S}L}{GI_{t}}=\frac{1}{{\left(\sqrt{k}+\frac{2}{\sqrt{k}}\right)}^{2}}\frac{M_{S}L}{GI_{t}}$$

Actually, in the design of MEMS torsional accelerometers, it’s important to get the maximum torsional angle $\phi_{S}\;$ by optimizing structure sizes to increase the mechanical sensitivity. In this paper, the condition of maximum torsional angle $\phi_{Smax}$ and it’s maximum value are:

(13) $$\sqrt{k}=\frac{2}{\sqrt{k}},k=2$$

(14) $$\phi_{Smax}=\frac{M_{S}L}{8GI_{t}}$$

This means that to achieve the maximum mechanical sensitivity, the lengths of both torsional beams’ part AB (CD) and BC should keep the relationship:

(15) $$l_{s}=2l_{e}$$

As Figure 5b presents, when the acceleration, a is applied along the sensing direction, the two pairs of torsional structures will rotate reversely. The torque to the geometrical center, *T*, and the moment equilibrium equation of the left torsional structure is:

(16) $$T=m_{4}al_{m4}-m_{1}al_{m1}=(m_{4}-m_{1})al_{m}=\Delta mal_{m}$$

(17) $$T=2M_{S}+F_{B}d$$

herein, Δm is the difference between the masses of the left torsional structure and $F_{B}$ is the force of *z*-axis component at the point B. According to the basic beam theory, the displacement of *z*-axis component at point B caused by $F_{B}$ can be calculated as:

(18) $$Z_{B}=\frac{7F_{B}L^{3}}{6144EI_{y}}$$

herein, *E* is the Young’s modulus of silicon, and $I_{y}$ is area moment of inertia of the front beam about the *y*-axis. Actually, the absolute value of the twist angle $\phi_{Smax}$ is extremely small in this structure, so the approximate calculation of the twist angle $\phi_{Smax}$ is:

(19) $$\phi_{Smax}\approx sin\phi_{Smax}=\frac{Z_{B}}{\frac{d}{2}}=\frac{7F_{B}L^{3}}{3072dEI_{y}}$$

(20) $$k_{\phi }=\frac{T}{\phi_{Smax}}=\frac{8\left(384d^{2}EI_{y}+14L^{2}GI_{t}\right)}{7L^{3}}$$

From the Equation (20), the torsional stiffness of the double differential torsional accelerometer based on the double-beam structure is related to the distance between two torsional beams d and the out-of-plane bending stiffness $EI_{y}$, which is different from the traditional double differential torsional structure based on single-beam. With the Taylor Series at the point $\phi_{Smax}=0$, the changed capacitances can be calculated as:

(21) $$\Delta C_{1}=\Delta C_{3}\approx \frac{ℰwl_{e}l_{m}}{d_{0}{}^{2}}\phi_{Smax}+\frac{ℰw\left(l_{e}{}^{3}+12l_{m}{}^{2}l_{e}\right)}{12d_{0}{}^{2}}\phi_{Smax}{}^{2}$$

(22) $$\Delta C_{2}=\Delta C_{4}\approx -\frac{ℰwl_{e}l_{m}}{d_{0}{}^{2}}\phi_{Smax}+\frac{ℰw\left(l_{e}{}^{3}+12l_{m}{}^{2}l_{e}\right)}{12d_{0}{}^{2}}\phi_{Smax}{}^{2}$$

(23) $$\Delta C=\left(\Delta C_{1}+\Delta C_{3}\right)-\left(\Delta C_{2}+\Delta C_{4}\right)=\frac{4ℰwl_{e}l_{m}}{d_{0}{}^{2}}\phi_{Smax}$$

where ℰ is the permittivity of the gas in the gap. Thus, the mechanical sensitivity of the double differential torsional capacitance accelerometer based on double-beam is obtained:

(24) $$S_{mech}=\frac{7ℰwl_{e}l_{m}{}^{2}L^{3}\Delta m}{2d_{0}{}^{2}\left(384d^{2}EI_{y}+14L^{2}GI_{t}\right)}$$

From Equation (24), the mechanical sensitivity of the double differential torsional accelerometer based on a double-beam is related to the distance between two torsional beams d, which is essentially different from the traditional double differential torsional accelerometer based on a single-beam. Also, with the decrease of the distance *d* between two torsional beams, the double-beam torsional stiffness will decrease from the Equation (20) and the mechanical sensitivity will rise under the same condition, which means that we can improve the mechanical sensitivity by changing the distance between two torsional beams when we pursue a smaller device area.

Actually, the novel device is much smaller than traditional double differential torsional accelerometers and the smaller device area will lead to a higher noise floor. Thus, it’s necessary to calculate the noise floor theoretically. The primary source of mechanical noise for the device is the Brownian motion of gas molecules surrounding the proof mass and the Brownian motion of the proof mass suspension on anchors. The theoretical mechanical noise of the accelerometer in this paper can be calculated with the equation [4]:

(25) $$TNEA=\sqrt{\frac{4K_{B}Tw_{n}}{QM}}$$

herein, Q is quality factor of the structure, M is the total mass of the proof masses in the accelerometer, $K_{B}$ is the Boltzmann constant, T is the absolute temperature and $w_{n}$ is the natural angular frequency. All the above parameters can be calculated easily based on Table 1 and basic theory. The calculated mechanical noise equivalent acceleration is about $0.13\;\mathrm{mg}/\sqrt{\mathrm{Hz}}$. From Equation (25), the proof mass can be enlarged to decrease the mechanical noise of this device.

## 3. Simulation Analysis

### 3.1. Simulation Analysis of Mechanical Sensitivity and Nonlinearity

**Figure 6:**
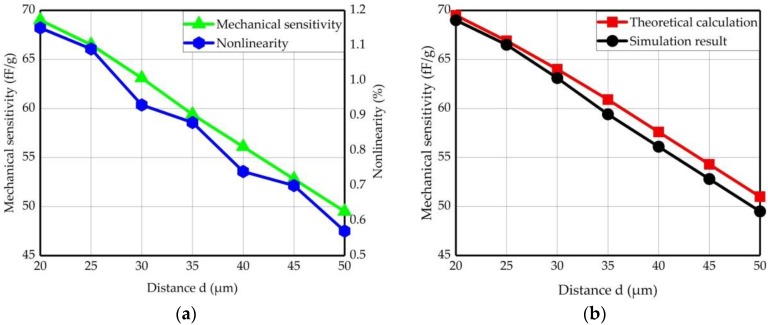
The simulation results of mechanical sensitivity and nonlinearity: (**a**) The simulation results of relationship between the nonlinearity and the mechanical sensitivity; (**b**) The theoretical calculation and simulation results of mechanical sensitivity.

Nonlinearity is the systematic deviation from the straight line that defines the nominal input-output relationship. The nonlinearity of the accelerometer can be calculated as:

(26) $$K={\frac{V_{j}^{*}-V_{j}}{\left|V_{max}-V_{min}\right|}|}_{max}$$

where $V_{j}$ is the average output voltage value of each acceleration, $V_{j}^{*}$ is the corresponding voltage value of on the fitting lines, $V_{max}$ and $V_{min}$ are the maximum and minimum voltages of output respectively. According to the Equation (2), the nonlinearity of accelerometer can also be calculated as:

(27) $$K={\frac{C_{j}^{*}-C_{j}}{\left|C_{max}-C_{min}\right|}|}_{max}$$

where $C_{j}$ is the average output capacitance value of each acceleration, $C_{j}^{*}$ is the corresponding capacitance value of on the fitting lines, $C_{max}$ and $C_{min}$ are the maximum and minimum capacitances of the output, respectively. As for the design of the novel double differential torsional capacitance accelerometer, it’s very important to balance the mechanical sensitivity and nonlinearity. In this paper, we establish a FEM model using COMSOL according to the dimensions in Table 1. As the nonlinearity is mainly related to the dimensions of the double-beam when we pursue a smaller device area, it’s necessary to perform the FEM analysis for the relationship between the mechanical sensitivity and nonlinearity of the measuring range (±15 g) by changing the distance between two torsional beams in the range of 20 μm to 50 μm under the same conditions. The simulation results are shown in Figure 6a. When the distance between two torsional beams *d* is decreased, the mechanical sensitivity will be increased. Also, the nonlinearity will be increased when the mechanical sensitivity is increased, which shows the importance of balancing the mechanical sensitivity and nonlinearity. Thus, the mechanical sensitivity and nonlinearity of the novel device are designed to be 59.4 fF/g and 0.88%, respectively, when the distance between two torsional beams *d* is 35 μm. In order to verify the correctness of the theoretical calculation, we compare the results of theoretical calculation in Equation (27) and our simulation. As shown in Figure 6b, the theoretical calculation and simulation results of mechanical sensitivity are in good agreement and the maximum relative error is 3% in the whole range of *d*, which demonstrates the theoretical calculation of mechanical sensitivity is sound.

### 3.2. Simulation Analysis of Shock Resistance

The post-impact response of the MEMS accelerometer has received attention in recent years [24,25]. In fact, shocks still represent an important issue as for the reliability of inertial MEMS devices and failures linked to cracks spreading in high stressed regions of the MEMS can suddenly occur [26]. Focusing on the physical effects of shocks and drops on the MEMS accelerometer, it can be shown that the mechanical side of the problem is by far the most prominent one and the electrical side can be disregarded [27]. This is due to the fact the inertial and possible contact forces turn out to exceed by orders of magnitude the electrical ones, when more than $10^{5}\;g$ is induced by the shock loading [24].

As far as the reliability of the sensor subject to shock-like loading is concerned, the value of the maximum principle stress $\sigma_{p}$ is considered the triggering factor for possible failure mechanisms [28]. Because of the re-entrant corners at the connection with the anchor and the substrate, $\sigma_{p}$ is actually located in critical regions very close to the end of the cross-sections of the supporting beams [29]. What turned out from previous experiments is that standard accelerometers can sustain high-g acceleration levels without malfunctioning, since the stress field in critical regions results to be much below the characteristic tensile strength of silicon (typically higher than 1 GPa) [27]. As for traditional single torsional beam structures, the critical regions are near the symmetry line of the differential masses, where the moment of force is the maximum. However, in the design of the double-beam structure presented in this paper, the position of critical regions can be moved away from the symmetry line of differential masses by increasing the distance between two torsional beams, which can reduce $\sigma_{p}$ and improve the shock resistance of the novel device. Thus, it’s necessary to perform a FEM simulation of the maximum principle stress $\sigma_{p}$ in critical regions by changing the distance between two torsional beams.

**Figure 7:**
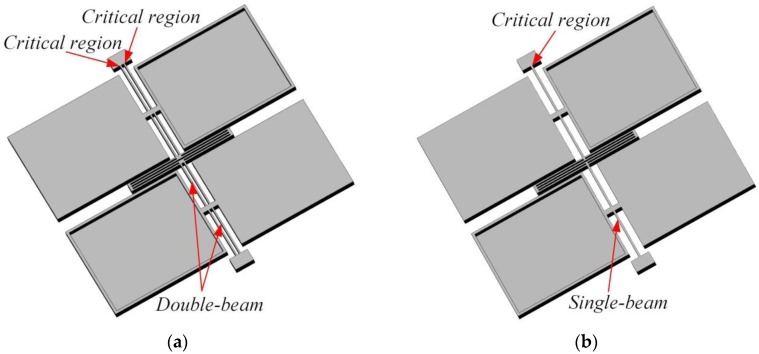
The simulation models of two accelerometers: (**a**) Simulation model of double-beam torsional accelerometer; (**b**) Simulation model of single-beam torsional accelerometer.

In this paper, we performed a FEM simulation for the novel device by changing the distance between two torsional beams *d* when a high-g input acceleration loading (with a peak value of about 5500 g) is applied along all three axes. The input acceleration loading is already considered and depicted in [30]. Meanwhile, the torsional stiffness remains the same by changing the width of single beam *b*, which means the mechanical sensitivity of the novel accelerometer remains at 59.4 fF/g. In order to make the results more comparable and meaningful, we have done the same FEM simulation for the single-beam accelerometer which has the same mechanical sensitivity. Considering the feasibility of fabrication processing and the accuracy of the mechanical sensitivity, the parameter *d* ranges from 25 μm to 45 μm and changes every 5 μm. Except for the torsional beams, the two structures are the same. The FEM simulation models of the two accelerometers are shown in Figure 7.

**Figure 8:**
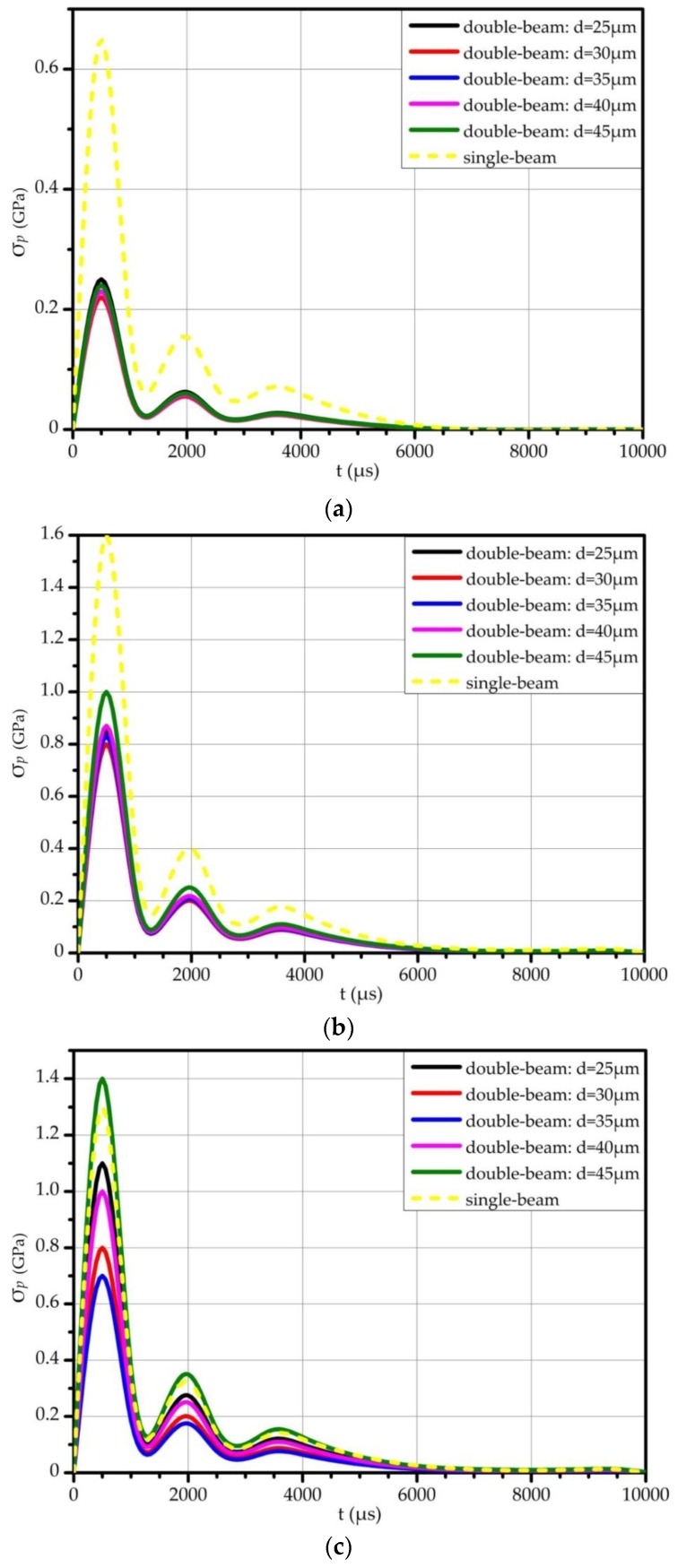
Time evolution of the maximum principal stress in critical regions of double-beam torsional accelerometer and single-beam torsional accelerometer when the high-g acceleration is applied along all three axes in trend line: (**a**) Simulation results when the high-g acceleration is applied along *x*-axis; (**b**) Simulation results when the high-g acceleration is applied along *y*-axis; (**c**) Simulation results when the high-g acceleration is applied along *z*-axis.

As the simulation results of the time evolution of the maximum principle stress in critical regions are not easily observed, we have selected 20 feature points of each line to draw the variation trend in Figure 8. As shown in Figure 8a,b, when the high-g input acceleration is applied along *x*-axis or *y*-axis, the double-beam structure has much lower $\sigma_{p}$ than the single-beam structure in the whole range of *d*. It can be seen from Figure 8c that the double-beam structure has lower $\sigma_{p}$ in a particular range of *d* when the high-g input acceleration is applied along *z*-axis. Thus, the double-beam structure can achieve lower $\sigma_{p}$ in critical regions and lower possibility of failure mechanisms than single-beam structure. In conclusion, this paper chooses the distance between two torsional beams as 35 μm to decrease $\sigma_{p}$ in critical regions and realize better shock resistance.

### 3.3. Simulation Analysis of Tolerance in Fabrication

Symmetry design is the key to the implementation of a double differential torsional MEMS accelerometer. However, fabrication imperfections are unavoidable in practical processing. A source of uncertainties which cannot be ignored at the film level is the variation in the over-etch [31]. In [32], an etch variation defect is one where the thickness of the device structure does not meet the design expectations due to the etch variations caused by fluctuations of temperature, etchant concentration and other reasons. Overall, a variation of about 10% in the geometry can be expected [33,34,35,36]. As for the novel device in this paper, the torsional double-beam is the key to the mechanical sensitivity, so it’s necessary to deliberately set some imperfections to analyze their influence to the mechanical sensitivity.

**Figure 9:**
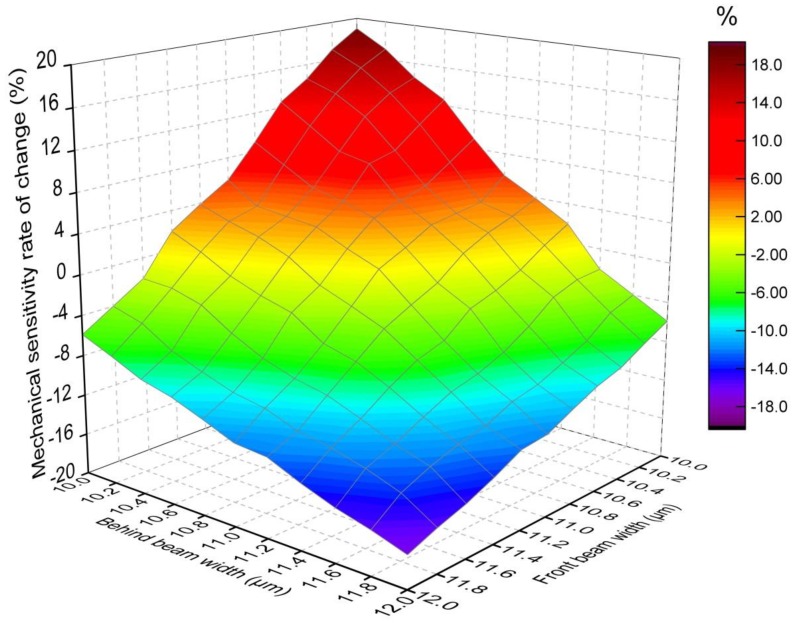
FEM simulation of the tolerance in fabrication by making the double-beam having different width.

Here, we perform a FEM analysis for the tolerance in fabrication by making the double-beam have different width. The novel device is set as a baseline and the range of machining error is set as ±1 μm. The simulation results are shown in Figure 9. They show that the effect of two beams’ widths on mechanical sensitivity rate of change is the same and the mechanical sensitivity deviation increases as the machining error increases. The mechanical sensitivity rate of change is from −4.2% to 3.6% when the maximum machining error is ±0.2 μm. However, when the maximum machining error is ±1 μm, the mechanical sensitivity rate of change is from −16.8% to 18.9%, which is a huge error for the novel device. Thus, in order to achieve mechanical sensitivity accuracy, the fabrication process should be designed carefully and the mask compensation should be implemented to decrease the machining error of the torsional beam.

### 3.4. Simulation Analysis of Temperature Robustness

**Figure 10:**
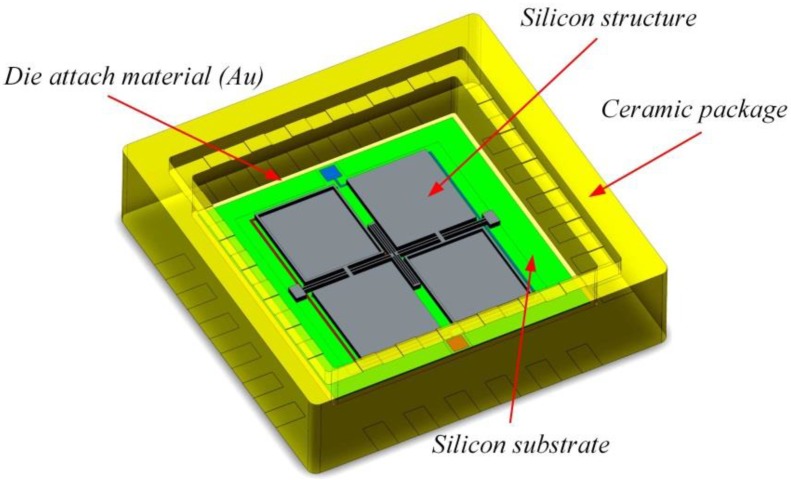
Schematic of the packaged device.

The temperature drift is one of the most important factors contributing to the change of the capacitance between the silicon plane and substrate. The proposed structure achieves the temperature self-calibration by the design of double differential configuration and symmetrical structure, greatly improving the temperature robustness of the device. The packaged device is shown in Figure 10.

**Figure 11:**
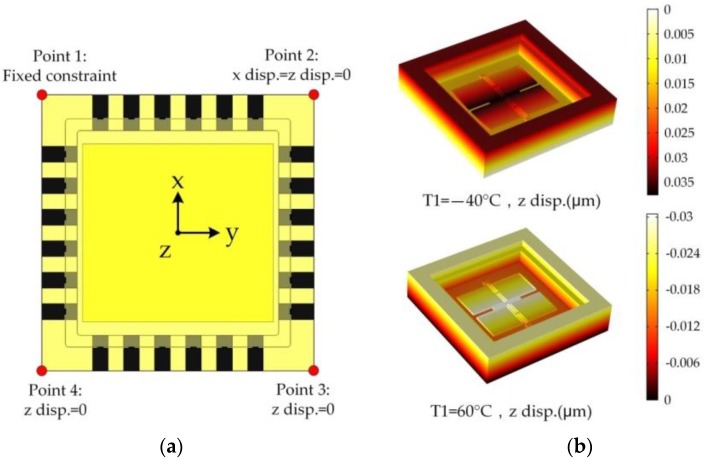

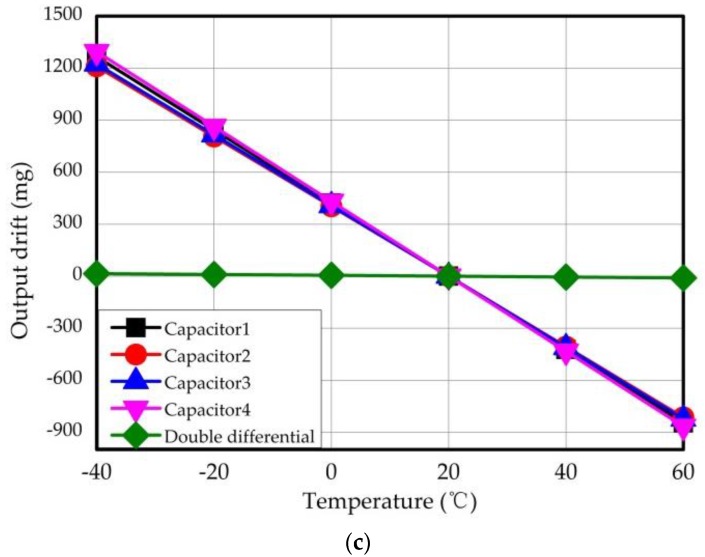
FEM simulation of the device at the temperature range from −40 ℃ to 60 ℃: (**a**) The constrain condition of the simulation; (**b**) The deformation at high and low temperature; (**c**) The simulation results of temperature draft.

The bonded accelerometer die, composed of a silicon structure and a silicon substrate, is attached on the ceramic package bottom by an Au film. As all the materials and connections will produce thermal stress with the temperature changes, the temperature performance simulation should be done for the whole system. In this paper, we have performed a FEM simulation of the device within the temperature range from −40 ℃ to 60 ℃ while the reference temperature is 20 ℃. The constraint condition is shown in Figure 11a. In order to make the whole system have free expansion, point 1 is fixed completely while the other points are constrained in special directions. The deformation at high and low temperature is shown in Figure 11b and the simulation results of temperature draft are shown in Figure 11c. It can be seen that, in the full temperature range, four capacitors have the same change trend. Also, each capacitor has about 2100 mg output draft which is a huge error for the MEMS accelerometer.

However, the design of double differential configuration in this paper eliminates the common mode variation signal of each capacitor by twice difference of two pairs of differential masses. Thus, we choose the double differential capacitance calculated by Equation (32) as the output in this paper and the temperature draft is reduced to 25 mg. The simulation results demonstrate the advantage of the double differential configuration in temperature self-calibration.

## 4. Fabrication Process

**Figure 12:**
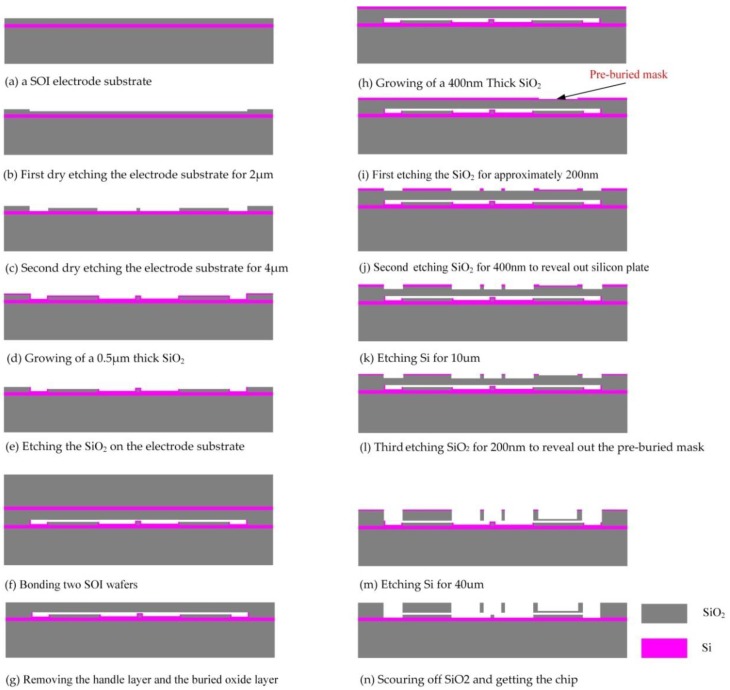
The fabrication process of the accelerometer.

For a traditional double differential torsional MEMS accelerometer, wet etching is utilized to fabricate the suspended silicon beam with an accurate sloping cross sectional shape [22,23]. However, the process enlarges the device area and reduces the utilization ratio of the silicon chip. In this paper, a novel process based on a pre-buried mask and dry etching techniques is used to fabricate the silicon structure, which makes the device more than 50 times smaller than a traditional double differential torsional MEMS accelerometer, greatly promoting the potential commercial value. The detailed processing procedure of the novel accelerometer is shown in Figure 12, where the process of the novel device is: (a) the starting material, a (100) silicon-on-insulator (SOI) wafer with device layer thickness of 6 μm; (b) first dry etching the electrode substrate for 2 μm to form the capacitive gap; (c) second dry etching the electrode substrate for 4 μm to form the silicon electrode; (d) growing of a 0.5 μm thick silicon dioxide layer; (e) etching the silicon dioxide on the electrode substrate; (f) bonding the SOI wafer of electrode substrate with a (100) SOI wafer with device layer thickness of 50 μm; (g) removing the handler layer and the buried oxide layer to fabricate to silicon structure; (h) growing of a 400 nm thick silicon dioxide; (i) first etching the silicon dioxide layer for half the thickness, approximately 200 nm, completing the pre-buried process in this step; (j) second etching silicon dioxide for 400 nm to etch the silicon structure; (k) first dry etching the silicon structure for 10 μm; (l) third etching of the silicon dioxide for 200 nm to reveal out the per-buried mask; (m) second dry etching the silicon structure for 40 μm; (n) scouring off the silicon dioxide to complete the fabrication.

As it shown in Figure 12, the fabrication process is mainly composed of three parts: the fabrication of the electrode substrate, the bonding process and the fabrication of the silicon structure. In order to reduce the alignment error caused by the bonding process in Figure 12f, the bonding process is designed before the fabrication of the silicon structure. Considering that the electrode substrate will be destroyed when the silicon structure is fabricated, the growing of a protection layer in Figure 12d is designed to protect the silicon structure. Also, we design the pre-buried dioxide process in Figure 12i to fabricate the differential silicon masses with different thicknesses.

## 5. Conclusions

This paper reports the design and simulation of a 4 mm^2^ double differential torsional MEMS accelerometer based on a double-beam configuration. The device area of the novel accelerometer is more than 50 times smaller than that of the traditional double differential torsional MEMS accelerometer. The design model is established and the working principle is analyzed in detail. Then the model of mechanical sensitivity is obtained theoretically to guide the structure design. Also, the nonlinearity, shock resistance and temperature robustness of the novel device are analyzed by FEM simulation to demonstrate the excellent features of the design. Afterwards, the fabrication tolerance is analyzed and the fabrication process is introduced.

Above all, the smaller device promotes the potential commercial value without sacrificing the specifications of mechanical sensitivity, nonlinearity and temperature robustness, which are 59.4 fF/g, 0.88% and 0.25 mg/℃ respectively. The mechanical noise is analyzed in theory and the calculated equivalent acceleration is about $0.13\;\mathrm{mg}/\sqrt{\mathrm{Hz}}$ in theoretical calculation. Compared with traditional single-beam silicon structure, the novel structure can achieve lower maximum principle stress in critical regions and reduce the possibility of failure mechanisms when high-g acceleration loading is applied along all three axes.

The next step of present investigation is to finish the manufacturing and testing of the novel device to verify the design method and simulation results reported in this paper. This approach is expected to provide meaningful guidance for the design of a novel device and the method can also be utilized in the design of other MEMS devices.

## Figures and Tables

**Table 1:** Geometrical parameters of the novel accelerometer.

Description	Symbol	Value
Wafer thickness	*t*	50 μm
Curved depth	*h*	40 μm
Width of single beam	*b*	11 μm
Distance between two torsional beams	*d*	35 μm
Effective area of each sensing capacitor	$A_{0}$	0.72${\;\mathrm{mm}}^{2}$
Original gap of the capacitor	$d_{0}$	2.0 μm
Original capacitance of each sensing capacitor	$C_{0}$	3.18 pF
Capacitance of reference capacitor	$C_{f}$	6.36 pF
Mechanical sensitivity	$S_{mech}$	59.4 fF/g
Fundamental resonance frequency	$f_{0}$	3269 Hz
Measuring range	$R_{a}$	±15 g
